# The interplay between noncoding RNAs and drug resistance in hepatocellular carcinoma: the big impact of little things

**DOI:** 10.1186/s12967-023-04238-9

**Published:** 2023-06-07

**Authors:** Yuan Fang, XiaoLi Zhang, HanFei Huang, Zhong Zeng

**Affiliations:** 1 Organ Transplantation Center, The First Affiliated Hospital of Kunming Medical University, 295 Xichang Road, Kunming, 650032 Yunnan People’s Republic of China; 2 Gastrointestinal and Hernia Surgery, The First Affiliated Hospital of Kunming Medical University, Kunming, Yunnan People’s Republic of China

**Keywords:** Hepatocellular carcinoma, Noncoding RNAs, Drug resistance, Targeted therapy, Immune therapy

## Abstract

Hepatocellular carcinoma (HCC) is the leading cause of cancer-related death in people, and a common primary liver cancer. Lacking early diagnosis and a high recurrence rate after surgical resection, systemic treatment is still an important treatment method for advanced HCC. Different drugs have distinct curative effects, side effects and drug resistance due to different properties. At present, conventional molecular drugs for HCC have displayed some limitations, such as adverse drug reactions, insensitivity to some medicines, and drug resistance. Noncoding RNAs (ncRNAs), including microRNAs (miRNAs), long noncoding RNAs (lncRNAs) and circular RNAs (circRNAs), have been well documented to be involved in the occurrence and progression of cancer. Novel biomarkers and therapeutic targets, as well as research into the molecular basis of drug resistance, are urgently needed for the management of HCC. We review current research on ncRNAs and consolidate the known roles regulating drug resistance in HCC and examine the potential clinical applications of ncRNAs in overcoming drug resistance barriers in HCC based on targeted therapy, cell cycle non-specific chemotherapy and cell cycle specific chemotherapy.

## Background

Hepatocellular carcinoma (HCC) accounts for around 90% of primary liver cancer cases and is associated with high mortality rates, as the sixth most prevalent cancer worldwide [1]. HCC is commonly associated with HBV or HCV infection, alcohol abuse, or nonalcoholic fatty liver disease (NAFLD) [2]. There are various therapeutic methods to treat pre-HCC, such as surgical resection, radiofrequency ablation, absolute ethanol injection, and chemoembolization [3]. Hepatic resection remains the first choice of treatment, even if it is associated with 70% of tumor recurrence and metastasis at 5 years [4]. Furthermore, systemic therapy is considered as the last line of defense for patients with postoperative recurrence, malignant vascular invasion, and extrahepatic spread, and for patients who are not application for operation [5]. The mainstay of cancer treatment is comprised of chemotherapy, immunotherapy, and targeted therapy [6]. Commonly employed therapeutic regimens for cancer treatment include drugs such as sorafenib (SOR), lenvatinib, regorafenib, 5-fluorouracil (5-FU), adriamycin (ADM), platinum-based chemotherapy, camptothecin, and gemcitabine. Meanwhile, Atezolizumab + Bevacizumab therapy, cabozantinib therapy have been administered recently [6–9]. However, drug resistance remains the primary restrictive factor to improve outcomes in patients with HCC [10].

Through multiple mechanisms of intrinsic and acquired drug resistance, hepatic tumor cells can avoid the cytotoxicity of chemotherapy and targeted therapy [10]. Drug resistance in cancer can be categorized into primary drug resistance and acquired drug resistance according to its mechanism. Primary resistance is when a cancer patient does not respond at all to the initial antitumor therapy, while acquired resistance is when a patient responds to the initial antitumor therapy, but the disease recurs or worsens after a duration of therapy [11, 12]. The effects of multidrug resistance (MDR) include enhanced drug efflux, elevated metabolism of xenobiotics, altered DNA repair capacity, growth, and genetic factors [13]. Each of these mechanisms could diminish the effect of treatment with administered drugs, inducing more difficulty in HCC systemic therapy. At the same time, due to the complexity of MDR mechanisms, there may never exist a single drug that can treat multiple cancers. Hence, gaining a more comprehensive comprehension of these mechanisms could aid in the formulation of novel approaches to target cancerous cells in the liver.

The majority of the genome generates noncoding RNAs (ncRNAs), which do not contain protein-coding instructions but rather produce noncoding transcripts that modulate gene expression and protein functionality [14]. Based on length and shape, ncRNAs can be generally classified into microRNAs (miRNAs), long ncRNAs (lncRNAs), and circular RNAs (circRNAs) [15]. Small, single-stranded, ncRNAs molecules known as miRNAs (20–24 nucleotides) are heavily involved in the post-transcriptional control of oncogenes and tumor suppressor genes, regulating their expression in various ways [16]. Furthermore, long noncoding RNAs (lncRNAs) are RNA molecules longer than 200 nucleotides, which influence gene expression by impacting proteins, RNAs, and DNA [17]. CircRNAs, a subtype of lncRNAs that from a covalently closed continuous loop without a 5'-3' or polyA tail, possess a relatively stable structure with highly tissue-specific expression in the eukaryotic transcriptome [18]. ncRNAs are useful molecular biomarkers and therapeutic targets, since they regulate molecules and drive or prevent oncogenic processes in diverse cancers [19]. Numerous ncRNAs have been shown to have vital roles in regular cellular processes as well as diseases such as cancer, and efforts are currently underway to translate these ncRNAs into clinical applications [20]. PCA3, which was the first biomarker to receive FDA approval, is a prostate-specific marker that is frequently overexpressed in prostate cancer. Its significance lies in the fact that it can be conveniently detected through noninvasive urine collection [21–23]. These ncRNA regulatory mechanism studies reveal the function of ncRNA in multiple cancers, which in turn provides new insight for scientists to develop specific cancer therapeutics, including on its part drug resistance.

Our review provides a systematic summary of the involvement of ncRNAs in drug resistance, including the underlying mechanisms, and their significance in current clinical practice. Subsequently, we discuss the significant potential of targeting ncRNA signaling via these underlying mechanisms in ncRNA biology to impact the systemic treatment of HCC.

## miRNAs and systemic treatment resistance

**Fig. 1:**
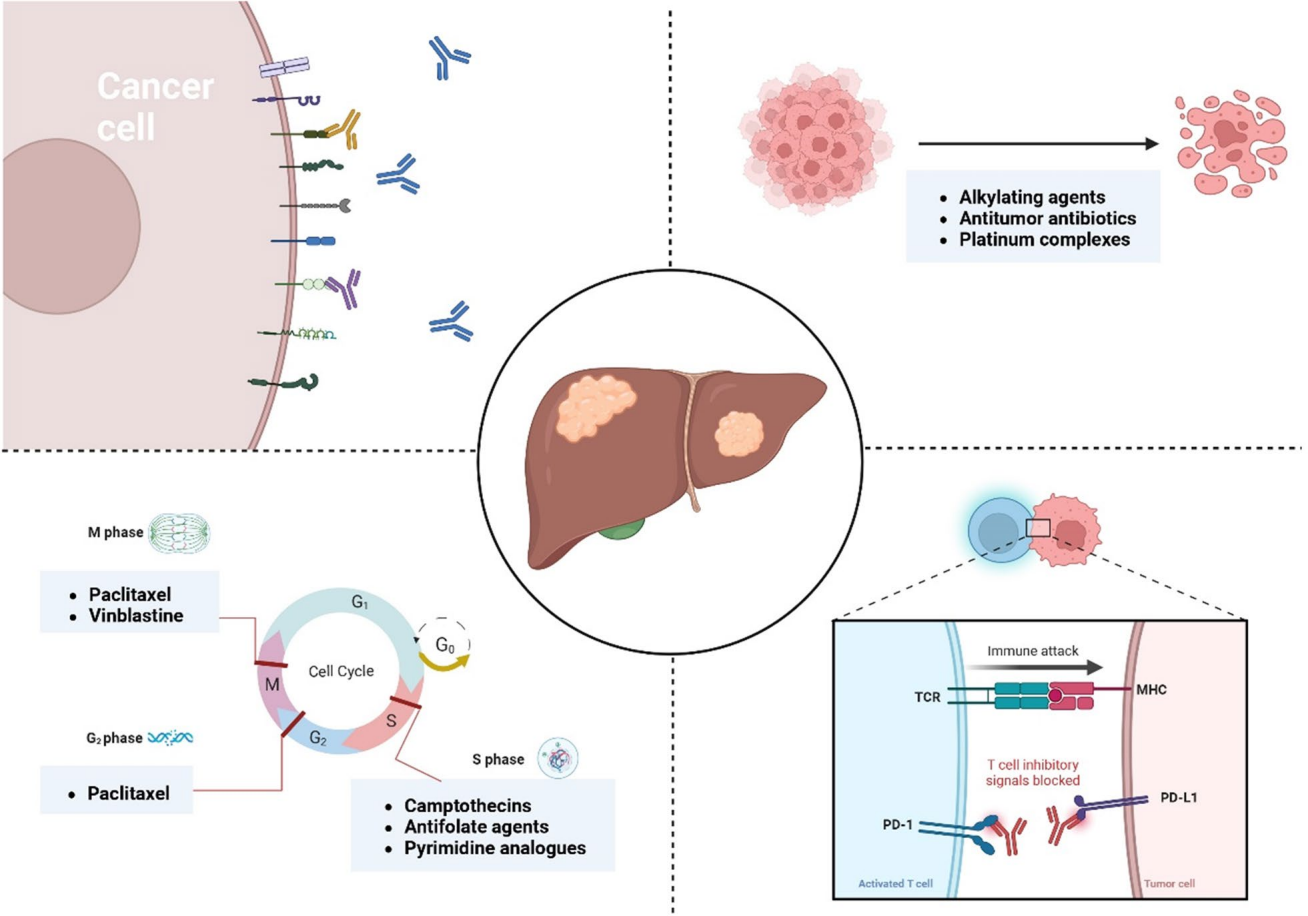
Systemic Treatment of HCC

At present, there are three clinical classification methods for antitumor systemic treatment, including chemotherapy (cell cycle nonspecific or specific of anti-HCC drugs), targeted therapy (SOR and lenvatinib), and immunotherapy (PD-1/PD-L1 checkpoint inhibitors) [24] (Fig. 1). Employment of long‐term cancer drug therapy easily results in drug resistance, especially in the systemic treatment of HCC, which is a tough challenge clinicians face [25]. miRNAs possess not only diagnostic and prognostic value as underlying markers, but also demonstrate therapeutic potential. As regulators of chemotherapy resistance, miRNAs hold promise as either biomarkers or therapeutic targets [26]. miR-155 was upregulated in gastric cancer and HCC and to play a tumor promoting role by mediating drug resistance in these tumors [27–29]. Numerous studies have indicated that miR-34 is a crucial miRNA tumor suppressor [30–32], and its target genes are involved in the drug resistance mechanism. Restoring low levels of miR-34 in drug-resistant tumor cells can effectively reestablish sensitivity to chemotherapeutic agents [33]. The discovery suggests that miRNAs could modulate drug resistance in HCC and miRNA-based therapeutic approaches are anticipated to enhance treatment efficacy for HCC patients.

The development of drug resistance is a multifactorial process, and the primary factors are the pathways and functions of the relevant miRNAs involved. Utilizing miRNA targeting to counteract particular malignant properties of cancer may have wider clinical ramifications, including the augmentation of sensitivity to treatment and suppression of resistance to systemic therapy [34]. Concurrently, abnormally expressed miRNAs, which function as oncogenes or tumor suppressor genes, has been implicated in the development and progression of many cancers, including HCC, offering novel perspectives on systemic therapy [35]. The primary role of miRNAs is to bind to the target 3'‐UTR of mRNA, thereby suppressing gene expression and influencing the malignant characteristics of HCC [36].

Exosomes, which range in diameter from 40 to 160 nm, are significant mediators of miRNAs [37]. Exosomes are linked to diverse bioactive molecules such as miRNAs, signaling peptides, lipids, and DNA [38]. An increasing number of studies have shown that exosomal miRNAs are implicated in the regulation of tumorigenesis, development, and drug resistance [39]. According to Fu et al., miR-32-5p was upregulated in HCC cells with MDR, and its expression had an adverse effect on PTEN in HCC samples [40]. Significantly, the transport of miR-32-5p by exosomes could activate the PI3K/Akt pathway, resulting in multidrug resistance in HCC [41]. miRNAs influence the occurrence, development, and drug resistance of HCC by regulating multiple genes. Compared with a single protein molecule, the regulation of miRNAs on diseases is more complex and comprehensive, which can improve the effectiveness and stability of drug therapy. The reversibility of miRNAs makes them different from other drugs. miRNAs can reverse drug resistance of HCC cells through direct regulation of gene expression. Therefore, miRNAs can be used as a strategy to reverse drug resistance and improve the effectiveness of treatment. At the same time, compared with other drugs such as chemotherapy, miRNAs have less toxic and side effects and less harm to patients, which can increase the tolerance of treatment. Moreover, miRNAs are tissue and disease specific and can be personalized according to the specific conditions of HCC patients. Based on the differences in miRNAs expression, precise targeted therapy can be performed to improve therapeutic efficacy and tolerance.

### MiRNAs and targeted therapy

In the treatment of liver cancer, miRNAs as potential targets of targeted therapy have attracted wide attention. However, drug resistance in HCC poses a challenge to the application of miRNAs targeted therapy. An in-depth understanding of the mechanism of miRNAs in HCC drug resistance can provide a theoretical basis for the development of miRNAs targeted therapy.

**Fig. 2:**
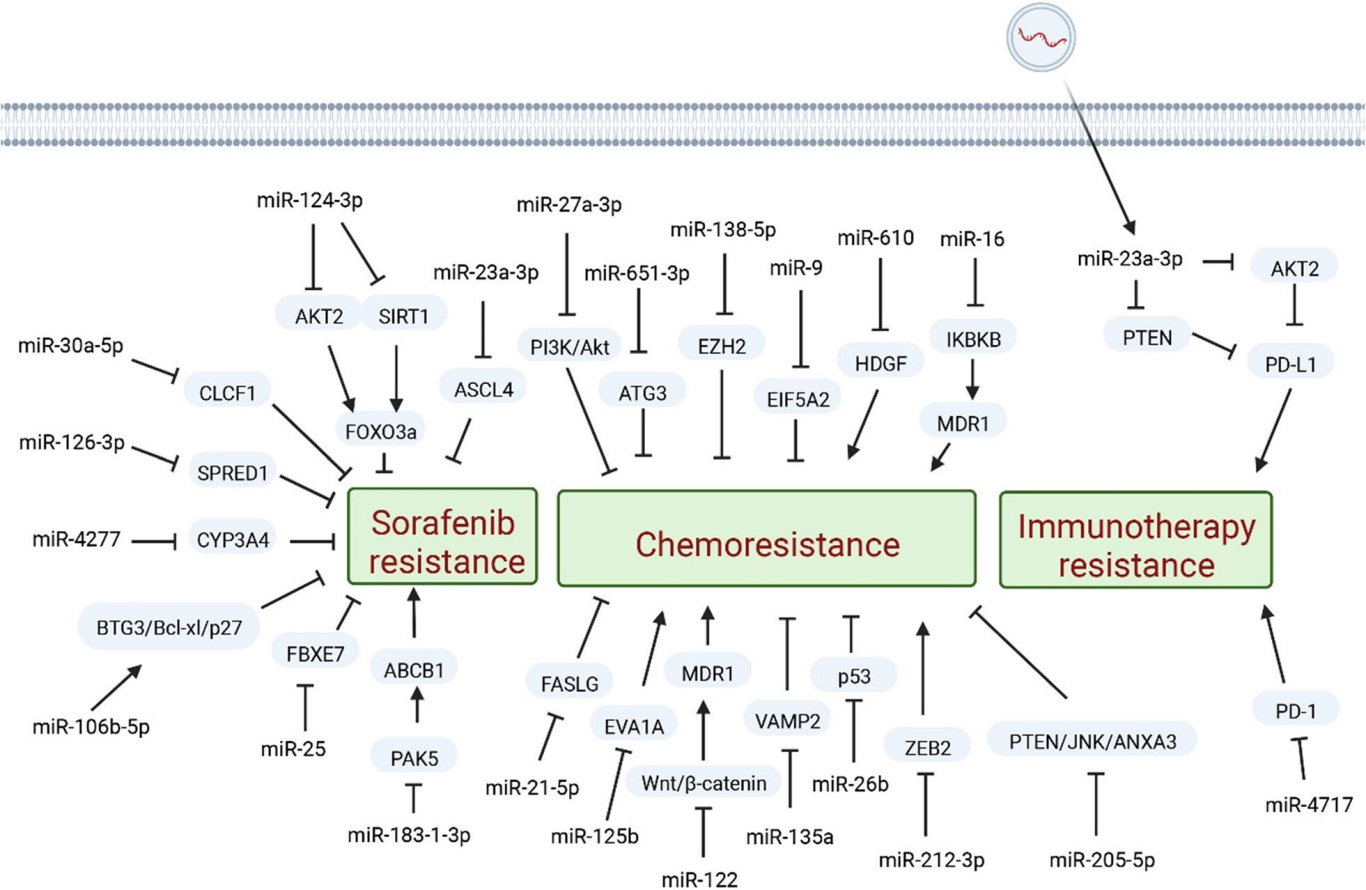
miRNAs and drug resistance in HCC. Arrows represent activation or production; blunt arrows represent inhibition

SOR is the first molecularly targeted drug approved by the FDA for the clinical treatment of HCC. It is a dual aryl urea multikinase inhibitor that induces apoptosis, inhibits angiogenesis, and suppresses tumor cell proliferation [42]. Immunocheckpoint inhibitors (ICI) such as nivolumab and pembrolizumab are approved for patients with advanced hepatocellular carcinoma who have already received SOR therapy. These drugs activate a patient's own immune system to attack tumor cells. One of its mechanisms of action is through the inhibition of the Raf/MEK/ERK signaling pathway, which can curb tumor proliferation. Additionally, SOR can inhibit the activity of vascular endothelial growth factor receptor (VEGFR), which helps to block tumor angiogenesis [43], and platelet-derived growth factor receptor (PDGFR) [44], resulting in indirect inhibition of tumor growth [45]. Therefore, this suggests that SOR has strong antitumor and antiangiogenic effects. Even though SOR is currently widely used, the side effects of SOR are its poor water solubility, fasting in scavenging metabolism, and inefficient tumor tissue absorption, which limits the efficacy of SOR [46]. Furthermore, some patients exhibit inherent resistance to SOR, while others develop acquired resistance after treatment. Even in cases where patients initially respond, resistance can rapidly emerge [47]. This is because SOR exerts antimetastatic and antiproliferative effects by many targets, including Raf, [48] EGFR, and PDGFR, but not all HCC tumors overexpress these targets [49]. Tumor heterogeneity can lead to suboptimal treatment efficacy because certain tumors do not depend on these pathways for tumorigenesis. As a result, acquired or primary SOR resistance represents a significant obstacle to patient survival in cases of HCC [50]. SOR is main type of targeted therapy for HCC without any additional clinical improvement before the introduction of lenvatinib, which was the result of its noninferiority to SOR approval. SOR was the central focus of trials, and all trials used it as a control to compare and evaluate new first-line drugs to improve outcomes in HCC patients [45]. As second-line therapies, regorafenib, cabozantinib, and ramucirumab have demonstrated survival benefits [51]. Recently, based on the evidence that miRNAs help for the therapeutic mechanisms underlying SOR resistance [52, 53] (Fig. 2).

Lenvatinib is a multitarget tyrosine kinase inhibitor used in the treatment of HCC. Studies have shown that miRNAs play an important regulatory role in the treatment of HCC with Lenvatinib. Some studies have shown that miR-3154 and miR-6071 are involved in the therapeutic effects of Lenvatinib in HCC cells [54, 55]. In conclusion, miRNAs play an important regulatory role in the treatment of HCC with Lenvatinib.

The ATZ/BEV regimen refers to the combination of Apatinib (ATZ) and Bevacizumab (BEV) for HCC. Studies have shown that miRNAs are involved in resistance mechanisms in ATZ/BEV treatment regimens and provide some useful therapeutic strategies. However, so far, little is known about the miRNA of ATZ/BEV in HCC resistance therapy, which is worth our further study.

Aberrantly expressed miRNAs, like miR-4277, miR-25, miR-138-1-3p, and others, can enhance SOR sensitivity [56–58]. miR-106b-5p can promote proliferation, metastasis and enhance SOR sensitivity by motivating the BTG3/Bcl-xL/p27 axis in HCC [59]. MiR-30a-5p can suppress SOR resistance by influencing CLCF1 directly in HCC. The miR-30a-5p/CLCF1/PI3K/AKT axis is a pathway targeting SOR to resist metabolism in HCC cells [60]. The occurrence of miR-124-3p.1 has been linked to early recurrence in HCC [61]. Research has demonstrated that the expression variations of miR-124-3p.1 are connected to the sensitivity of SOR, and overexpression of this molecule can enhance SOR-induced apoptosis [62].

However, aberrantly expressed miRNAs can weaken SOR sensitivity [63]. The resistance of HCC cells to SOR was found to be heightened when there is a disturbance in miR-126-3p. It was discovered that miR-126-3p/SPRED1 modulates the ERK signaling pathway, thereby influencing SOR sensitivity in HCC [64].

Aberrant expression of miRNAs, especially due to epigenetic changes, is one of the critical role regulating SOR resistance of HCC [65]. Generally, the miR-23a-3p aberrant expression is found in HCC and is correlated with epigenetic changes, that lead to dysregulation of the apoptosis, cell cycle, invasion, migration, and immune response [66]. However, the aberrantly expressed of miR-23a-3p, epigenetic repressor of ferroptotic cell death in HCC, can be induced by SOR. The disruption of miR-23a-3p has a significant impact on the response of HCC and the effectiveness of SOR treatment. MiR-23a-3p by influencing iron overload and lipid peroxidation dysregulate SOR-induced ferroptosis [67]. In the future, it is necessary to explore possible therapeutic combinations targeting miRNAs with SOR for improved treatment of HCC patients [67].

The combination of MiRNAs targeted therapy with other therapies (such as chemotherapy and radiotherapy) can reduce the drug resistance of HCC and improve the therapeutic effect. A multi-target treatment strategy was adopted. Due to the complex mechanism of drug resistance inHCC, single miRNA-targeted therapy may not be able to completely solve the problem. Therefore, adopting multi-target treatment strategy may be an effective approach. To study the dose and medication regimen of miRNAs targeted therapy. The dose and medication regimen of miRNAs targeted therapy have important influence on the therapeutic effect and drug resistance, and should be personalized according to the specific situation of the patient.

### MiRNAs and chemotherapy

According to their effects on cell proliferation kinetics, antitumor chemotherapeutic drugs are generally classified into cell cycle-nonspecific and cell cycle-specific drugs, which is extremely informative for rational clinical drug use [68]. Platinum, antibiotics, and alkylating agents are the most widely used clinically as representatives of cell cycle nonspecific chemotherapy drugs [69–71].

The discovery of ncRNAs has shed new light into platinum-related genes and may provide reliable predictive biomarkers by regulating resistance to chemotherapy drugs [72]. Although it had been proven up to the now that cisplatin, the drug licensed in 1978, is one of the most successful chemotherapy drugs in the world, the drug is now facing a serious challenge because its drug resistance leads to a bottleneck in clinical application [73]. MiRNAs are associated with the regulation of cisplatin resistance [74]. Upregulation of of miR-27a-3p can significantly enhance the rate of inhibition and apoptosis induced by cisplatin treatment, as it regulates the PI3K/Akt pathway [75]. Furthermore, miR-651-3p can inhibit ATG3 [76] mediated cell autophagy to strengthen the susceptibility of HCC to cisplatin resistance [77]. Studies have revealed that miR-138-5p inhibits EZH2 and intensifies the sensitivity of HCC to cisplatin [78]. Likewise, the overexpression of miR-9 in HCC increases their sensitivity to cisplatin by preventing EMT and inhibiting EIF5A2. This sheds light on how miR-9 regulates HCC chemosensitivity and provides a potential target for enhancing the effectiveness of HCC chemotherapy [79]. Apart from its role as a tumor suppressor in HCC, miR-610 also affects the resistance of HCC cells to cisplatin by specifically silencing the HDGF gene [80]. Conversely, the targeting of FASLG by miR-21-5p decreased the sensitivity of HCC to cisplatin treatment [81].

Following surgical resection, oxaliplatin (OXA), a third-generation platinum compound, is among the favored chemotherapeutic drugs and is administered as a maintenance therapy for patients with advanced HCC. Resistance to OXA is a huge challenge [82]. There are still underlying clinical benefits of OXA therapy, and because of the lack of biomarkers, patients with OXA-resistant HCC are always overlooked [83]. A correlation has been reported between low expression of miR-125b and OXA resistance. When miR-125b represses proliferation, invasion, and EMT, its expression is elevated in HCC cells that are resistant to OXA. This suggests that miR-125b may heighten cellular sensitivity to OXA by reducing autophagy mediated by EVA1A [84]. Moreover, Elevated levels of miR-122 were found to enhance the sensitivity of HCC to OXA, resulting in increased apoptosis of HCC cells [85]. The first-line drugs in the present clinical setting, OXA has considerable application prospects, while research on miRNAs is far less advanced than that on cisplatin.

Over the past years, there has been an expansion of knowledge regarding the role of antibiotics in health and disease, including HCC [86]. Antibiotics [87], such as adriamycin (also known as doxorubicin), which are a significant class of medications, can cause DNA damage and trigger the relevant signaling pathway. This can lead to the activation of powerful anti-apoptotic genes and, in turn, result in chemoresistance of cancer cells [88]. Because of its finite tissue specificity, especially the mechanism of free radical generation and lipid peroxidation, doxorubicin (DOX), as one of the most frequent anticancer drugs, leads to chemotherapy resistance and tumor recurrence [89]. Several studies have shown that miR-135a can induce chemoresistance in certain cancers, which miR-135a-5p targets VAMP2 and has the ability to counteract the apoptosis induced by Dox in HCC [90, 91]. The current study proves the function of miR-26b in DOX chemoresistance, and the expression of miR-26b is downregulated in HCC cells. Additionally, treatment with a miR-26b mimetic in combination with doxorubicin strengthens the HCC sensitivity to DOX [92]. The expression of miR-200a is suppressed in both human HCC and HCC tumor cell lines. Its upregulation restrains the growth of HCC and strengthens the antitumor effect of doxorubicin in HCC by directly modulating tumor metabolism and autophagy [93].

Cell cycle-specific anti-HCC drugs mainly consist of plant antitumor drugs and antimetabolites [94, 95]. Plant antitumor drugs primarily act on the mitotic phase and the cell cycle of M phase. One of the representative drugs used for anti-HCC is paclitaxel, which includes paclitaxel (PTX) [96] and docetaxel (DOC) [97]; Vinblastine [98], such as vincristine (VCR) and vinorelbine (NVB); camptothecins, such as hydroxycamptothecin (HCPT) and irinotecan (CPT-11), can be used as adjuvant chemotherapy for HCC or for the treatment of drug-resistant HCC. As a common drug for HCC cell cycle-specific chemotherapy, PTX is a naturally occurring tricyclic diterpenoid compound that is synthesized in the bark. Because PTX is blocked by its low drug-fast, it is important to find the underlying molecular mechanisms to enhance PTX-sensitivity [99]. Additional research is needed in preclinical and clinical studies to explore factors that contribute to PTX resistance, such as mutations in ABC transporters and miRNAs, as well as the side effects of PTX. These side effects include peripheral neuropathy and hypersensitivity reactions that are associated with the vectors used to overcome its poor solubility [96, 100]. Current research has shown that miR16 increases chemoresistance to PTX in HCC [101]. In addition, miR-212-3p has low expression of HCC with PTX-resistant and can decrease PTX resistance by targeting ZEB2 to influencing EMT, migration and invasion [102].

By using compounds such as the normal physiological metabolic structure of the human body, antimetabolites can be involved in cellular physiological functions, disturb the function of normal metabolites, and block the normal metabolism and growth of tumor cells [103]. Antimetabolites commonly used in chemotherapy include folic acid antagonists, purine analogs, and pyrimidine analogs. One of the main drugs used to treat HCC is 5-FU, [104], capecitabine (CAPE) [105], GEM, etc., all of which are pyrimidine analogs. They induce antitumor activity by inhibiting tumor cell nucleic acids and regulating the body's immune response, in which CAPE is converted into 5-FU after being metabolized by liver and tumor tissue [106, 107]. Clinically, 5-FU is usually combined with OXA, which plays an ideal role in FOLFOX [108]. At the same time, strategies that combine GEM with TACE or OXA have fewer adverse drug reactions in treatment of advanced HCC, while both show obvious drug resistance. 5-FU is an antimetabolite drug that is commonly used to treat various types of cancer, including HCC [109]. 5-FU is an antimetabolite drug that is commonly used to treat various types of cancer, including HCC [110]. Studies have shown dysregulation of the oncogenic miR-145 in drug-resistant HCC, but upregulation of miR-145 expression can inhibit the proliferation and promote apoptosis of drug-resistant HCC cells [111].

### MiRNAs and immune therapy

Immunotherapy, including therapies targeting programmed death 1 (PD-1), [112] programmed death ligand 1 (PD-L1), [113] and cytotoxic T lymphocyte antigen-4 (CTLA-4), [114] has revolutionized cancer treatment and resulted in remarkable clinical responses in various solid tumors, including HCC. [115] At the same time, ICI are also a hot research topic in lncRNAs. The clinical efficacy of anti-PD-1/PD-L1 therapy can be attributed mainly to the reactivation of tumor antigen-specific T cells that are suppressed by the interaction of PD-1 on T cells with its ligand PD-L1 on tumor cells [116]. However, the therapy as a single agent has a low response rate of only around 10% to 30% of patients, which is not considered satisfactory [117, 118]. PD-1 and PD-L1 resistance include both ‘primary resistance’ and ‘acquired resistance’ [119]. In addition, there is evidence that miRNAs can regulate the expression of PD-1 and PD-L1, which are important for tumor immune escape and the creation of a microenvironment that supports tumor growth and development [120]. The expression of PD-1 can be adjusted allele-specifically by miR-4717 through various interactions with its polymorphic target in the PD-1 3’ UTR [121]. Furthermore, high-throughput sequencing analysis of exosomes has revealed their potential role in strengthening the expression of PD-L1 and inflammatory cytokines in macrophages. This, in turn, leads to a decrease in the CD8 + T-cell ratio and an increase in T-cell apoptosis, which contributes to the generation of a tumor-favorable microenvironment. Exosomes have been found to play a role in the regulation of PD-L1 expression by transferring miR-23a-3p, which in turn regulates PTEN and AKT [122].

Undoubtedly, the potential of miRNAs is immense, and their significant role in drug resistance could serve as promising circulating biomarkers as well as future surrogates for predicting therapeutic response. However, some additional evidence must be provided before miRNAs can enter routine clinical protocols.

## LncRNAs and systemic treatment resistance

Most expressed transcripts do not code for protein, with those > 200 nt in length being broadly classified as lncRNAs, which have certain traits they share with mRNAs [123]. Given their mutual effects, lncRNAs in the nucleus play a different role than those in the cytoplasm [124]. LncRNAs have diverse functions depending on their subcellular location. Those located in the nucleus can affect transcription through chromatin interactions and remodeling. On the other hand, in the cytoplasm, lncRNAs are involved in mediating signal transduction pathways, translational programs, and posttranscriptional control of gene expression [125] (Fig. 1).

### LncRNAs and targeted therapy

Several methods for developing resistance to SOR have been identified, such as decreased drug uptake, increased intracellular drug metabolism, enhanced drug excretion, alterations in molecular targets that impact pathway activation or inactivation, changes in DNA repair mechanisms, protein dysfunction in the cell cycle, and modulation of the tumor microenvironment [126]. Upregulation of lncRNA FAM225A [127] was found in HCC and HepG2/SOR cells with SOR-resistant, and the resistance of HepG2/SOR to SOR was inhibited by FAM225A. FAM225A was discovered to interact with miR-130A-5p to downregulate CCNG1 expression, leading to the promotion of SOR resistance and inhibition of cell apoptosis. This indicates that lncRNAs can influence SOR resistance in terms of apoptosis [128]. The downregulation of the lncRNA KCNQ1OT1 has been shown to decrease PD-L1 expression, reverse SOR resistance, and overcome immune evasion through miR-506 in both HCC tissues and cells that are resistant to SOR [129]. NIFK-AS1 knockdown has been found to sensitize HCC cells to SOR by reducing the uptake of SOR and inhibiting the drug transporters OATP1B1 and OATP1B3. High levels of NIFK-AS1, on the other hand, can contribute to the desensitization of HCC cells to SOR. Clinical trials have indicated that HCC patients with low NIFK-AS1 levels respond better to SOR treatment, while those with high NIFK-AS1 levels do not. In vivo studies using PDX models have also confirmed that lower levels of NIFK-AS1 are associated with better therapeutic efficacy of SOR [130]. Increased expression of lncRNA HANR in HCC by sponging with miR-29b, inhibited its expression, affected its target protein expression, autophagy-associated protein 9A antibody, and ultimately enhanced resistance to autophagy-associated SOR [131].

### LncRNAs and chemotherapy

Silencing LINC01234 and upregulating miR-31-5p was found to inhibit MAGEA3 expression, leading to a more malignant phenotype in HCC cells and increased sensitivity to cisplatin-induced apoptosis. Furthermore, knockdown of MAGEA3 resulted in decreased resistance of HepG2 cells to cisplatin by reducing MRP2, MRP3, and MDR-1 expression [132]. The expression of lncRNA TPTEP1 was found to be significantly reduced in HCC cells through RNA-seq analysis of differential gene expression. High levels of TPTEP1 have been shown to increase the sensitivity of hepatocellular carcinoma cells to apoptosis induced by cisplatin; otherwise, its overexpression led to increased neoplasia and invasion of HCC [133]. The enhanced cisplatin sensitivity of cisplatin-resistant HCC cells was observed following knockdown of LINC00173. A direct interaction between LINC00173 and miR-641 was confirmed through a luciferase reporter assay, and the inhibition of miR-641 reversed the effects of LINC00173 knockdown on cisplatin sensitivity in HCC cells [134]. TINCR played a key role to miR-195-3p which competitive endogenous sponge, alleviating its inhibition of ST6GAL1 and activating κB signaling pathway. Animal assays confirmed that downregulated of TINCR decreased tumor progression and OXA resistance [135].

Silencing LINC01134 induced ferroptosis in HCC, thereby promoting the sensitivity of hepatocellular carcinoma cells to OXA [136]. Meanwhile the activation of the antioxidant pathway via the transcription factors SP1 to P62 by LINC01134 was shown to influence cell viability, apoptosis, and mitochondrial homeostasis, resulting in OXA resistance both in vitro and in vivo. In patients with HCC, the expression level of LINC01134 was found to be positively correlated with the levels of P62 and LSD1, and the level of SP1 was positively correlated with P62 [137]. A potential approach to overcome OXA resistance in untreated patients may involve targeting the LINC01134/SP1/P62 axis.

Knockdown of HOTAIR inhibited the HepG2/Taxol and SMMC7721/Taxol invasion and apoptosis by upregulating miR-34a, when downregulating miR-34a promote the resistance of paclitaxel, showing that HOTAIR was relevant with miR-34a [138]. HCC tissues exhibited significant upregulation of LINC00680, and high levels of LINC00680 were associated with poor prognosis in patients. Overexpression of LINC00680 led to enhanced stemness of HCC cells and reduced chemosensitivity to 5-FU, both in vitro and in vivo. In contrast, knockdown of LINC00680 had the opposite effect [139]. HCC tissues exhibited significant upregulation of LINC00680, which was associated with poor prognosis in patients with high LINC00680 levels. Increased LINC00680 expression significantly increased the stemness of HCC cells and decreased their sensitivity to 5-FU treatment both in vitro and in vivo, while reducing LINC00680 expression had the opposite effect [140]. These findings contribute to our understanding of how lncRNAs contribute to HCC progression and, more importantly, provide insights into potential strategies for overcoming chemoresistance in HCC. Overexpression of lncRNA AY and SNHG16 can modulate 5-fluorouracil resistance and impact tumor development, metastasis, and angiogenesis in animal models [141, 142].

### LncRNAs and immune therapy

**Fig. 3:**
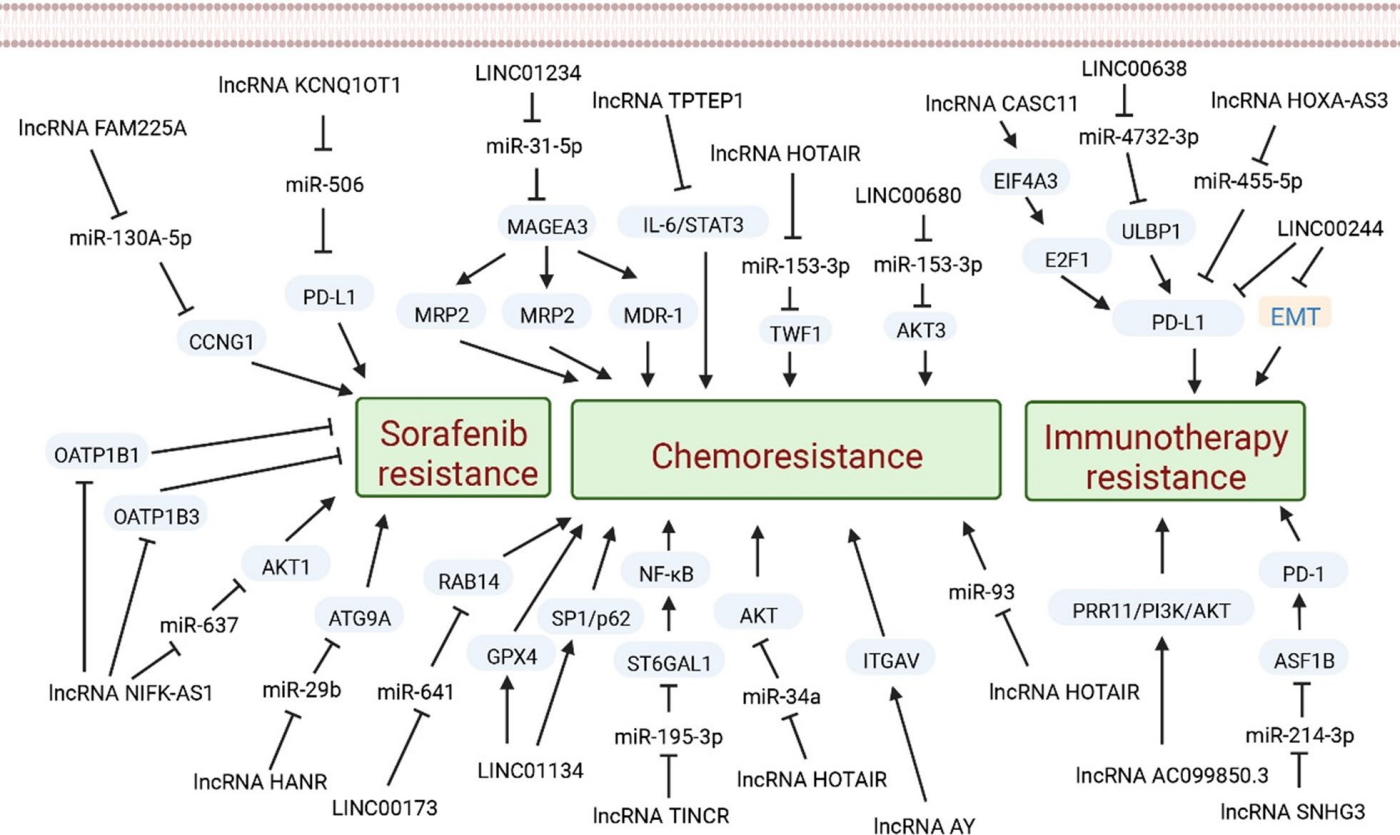
LncRNAs in HCC with drug resistance. Arrows represent activation or production; blunt arrows represent inhibition.

PD-1 has been shown to suppress the immune response by regulating T-cell activation and function, which can result in tumor invasion and postoperative recurrence in patients with HCC [143]. LncRNA SNHG3 has been confirmed to promote the level of PD-1 via changing ASF1B in HCC [144]. Moreover, the SNHG3/miR-214-3p/ASF1B axis plays a role in HCC recurrence by promoting immune tolerance and escape, with PD-1 being a key factor in this process [145]. Although PD-L1 has an inhibitory effect on the antitumor immune response has gained widespread attention, recent studies indicate that PD-L1 has a significant part in tumorigenesis and drug resistance. The observed positive correlation of AC099850.3 with key immune checkpoint molecules (PD-L1, PD-L2, PD-1, and CTLA4) has made AC099850.3 a potential HCC immunotherapy target [146]. LncRNAs play a role in various epigenetic regulatory mechanisms. By downregulating PD-L1 expression, LINC00244 inhibits HCC proliferation, metastasis, and invasion, and can serve as a predictor of HCC clinical outcomes [147]. Compared to normal hepatocytes, the expression of PD-L1 is elevated in HCC. The effects of HOXA-AS3 shRNA on the proliferation, invasion, and colony formation of HCC cells were reversed by the overexpression of PD-L1 [148]. Tumor and peritumoral samples exhibited PD-L1 expression, and in most HCC cases, PD-L1 expression was upregulated. Meanwhile, colocalization analysis showed that PD-L1 and ULBP1 were co-expression. The ceRNA network of ULBP1 was strongly correlated with TMB, and PD-L1 might play a crucial role in immune evasion in HCC with high TMB [149]. Aberrant expression of PD-1/PD-L1 regulated by lncRNAs may contribute to a poor prognosis of HCC through various molecular mechanisms. LncRNA CASC11 shows that awaken the NF-κB and PI3K/AKT/mTOR axis and therewith influence PD-L1 through E2F1 upregulation, thereby promoting proliferation, migration, and glucose metabolism of HCC [150]. At present, lncRNA and immune therapy for HCC are mainly the focus of preclinical studies, and there are a few clinical studies. (Fig. 3).

## CircRNAs and systemic treatment resistance

CircRNAs, a novel type of intrinsic RNAs, are closed circular structures covalently [151]. Multiple functions have been identified for circRNAs, which can act as protein scaffolds or miR sponges, thereby reducing the ability of miRNAs to target mRNAs [152]. Recent studies have revealed that circRNAs can function as ceRNAs, regulating PD-L1 expression and impacting tumor immune evasion in various types of cancer [153]. The mechanism of immune escape involves various factors such as impaired antigen presentation, altered cell death processes, metabolic abnormalities, and modulation of immunosuppressive cell populations and aberrant cytokine production [154]. Emerging data suggested that circRNAs would influence immunosuppression and curative effect of anti-PD-1 in HCC. The overexpression of circMET in tumors resulted in decreased levels of CXCL10 and reduced infiltration of CD8 + T cells, as compared to control tumors [155]. CircMET, a tumor circRNA, promotes immune tolerance via Snail/DPP4/CXCL10 axis [156]. CircUHRF1 promotes a progression of HCC and immunosuppression in the NK-cell-dependent type. CircUHRF1 suppresses the activity of NK cells by modulating miR-449C-5p and regulating the level of TIM-3. Subsequently, circUHRF1 drives anti-PD1 immunotherapy with resistance [157]. For circRNA, dysregulated circKCNN2 influences HCC recurrence and enhances the sensitivity of lenvatinib by miR-520c-3p/MBD2 [158]. In general, circRNAs are expected to open a new window for tumor immunotherapy.

## Conclusions

The issue of drug resistance represents a major challenge in the clinical management of HCC and requires urgent resolution. Intrinsic and acquired drug resistance are major impediments to cancer treatment [159]. In this review, we provide an overview of preclinical studies on the mechanisms of drug resistance in HCC. However, there is still a long way to go before practical application. First, dysregulation of ncRNAs has demonstrated potential capabilities for the regulation of systemic therapeutic resistance. Understanding the main characteristics which drug resistance can help to discover new therapies to overcome drug-resistant HCC, and targeting these dysregulated miRNAs, lncRNAs and circRNAs could be a hopeful therapeutic target to remedy drug resistance. Treatments targeting these abnormally expressed ncRNAs are a significative way to therapy drug resistance. In addition, nucleic acid drugs targeting nanocarriers and initiating and enhancing antitumor responses have come of age in the context of biocompatibility and cell type development [160]. Exogenous expression of tumor suppressor ncRNAs can be used to knock down oncogenic ncRNAs through small interfering RNAs [161] or shRNAs [162] which has been studied for its potential in reversing drug resistance in HCC. FDA and EMA have approved more than a dozen nucleic acid therapies for rare and genetic diseases, and there are currently dozens of registered clinical trials exploring the potential of these therapies for various cancers [163–166]. However, ncRNAs are readily degraded in the internal environment and cannot enter the cell interior on their own, making it difficult to achieve lasting therapeutic effects. RNA-based drug delivery remains one of the greatest challenges for RNA therapies, which are often applied in clinical practice using chemical modification to add the targeting moiety [167, 168]. With the development of nanotechnology and the invention of various multifunctional nanosystems, RNA can be delivered efficiently to specific sites of action. The current body of evidence indicates that ncRNAs play a causal role in drug resistance, but the mechanisms underlying this phenomenon are still being uncovered. Finally, we lack biomarkers for drug resistance in HCC. Understanding the molecular mechanisms underlying anticancer drug resistance is crucial for the development of novel precision medicine treatments that can overcome specific and well-defined mechanisms of drug resistance. And the treatment of HCC needs to be individualized according to the specific conditions of the patient, and the therapeutic effect and the quality of life of the patient should be comprehensively considered (Tables 1 and 2).

**Table 1:** miRNAs and drug resistance in HCC

miRNA	Expression	Target	Drug	References
miR-32-5p	Upregulated	PTEN and PI3K/Akt	Multidrug	[40]
miR-106b-5p	Upregulated	BTG3/Bcl-xL/p27 axis	SOR	[59]
miR-30a-5p	Upregulated	CLCF1/PI3K/AKT axis	SOR	[60]
miR-126-3p	Upregulated	SPRED1	SOR	[64]
miR-10b-3p	Upregulated	Cyclin E1	SOR	[169]
miR-25	Downregulated	FBXW7	SOR	[58]
miR-124-3p.1	Downregulated	FOXO3a	SOR	[62]
miR-138–1-3p	Upregulated	PAK5	SOR	[57]
miR-15a-5p	Upregulated	eIF4E	Pirarubicin	[170]
miR-4461	Upregulated	SIRT1	Cisplatin	[171]
miR-27a-3p	Downregulated	PI3K/Akt	Cisplatin	[75]
miR-651-3p	Upregulated	ATG3	Cisplatin	[76]
miR-138-5p	Upregulated	EZH2	Cisplatin	[78]
miR-9	Upregulated	EIF5A2	Cisplatin	[172]
miR-610	Downregulated	HDGF	Cisplatin	[80]
miR-21-5p	Upregulated	FASLG	Cisplatin	[81]
miR-125b	Downregulated	EVA1A	OXA	[84]
miR-122	Downregulated	Wnt/β-catenin	OXA	[85]
miR-135a	Upregulated	VAMP2	DOX	[90, 91]
miR-26b	Downregulated	USP9X	DOX	[92]
miR-145	Upregulated	TLR4	5-FU	[111]

**Table 2:** lncRNAs and drug resistance in HCC

lncRNA	Expression	Target	Drug	References
HCG18	Upregulated	GPX4	SOR	[173]
LINC01273	Upregulated	miR-600/METTL3	SOR	[174]
FAM225A	Upregulated	miR-130A-5p/CCNG1 axis	SOR	[127]
KCNQ1OT1	Upregulated	miR-506/PD-L1 axis	SOR	[129]
NIFK-AS1	Upregulated	OATP1B1 and OATP1B3	SOR	[130]
HANR	Upregulated	miR-29b/ATG9A axis	SOR	[175]
LIMT	Downregulated	miR-665	SOR	[176]
PURPL	Upregulated	P53	Doxorubicin	[177]
ARSR	Upregulated	Akt/NF-κB	Doxorubicin	[178]
PLAC2	Upregulated	miR-96	Cisplatin	[73]
LINC01234	Upregulated	miR-31-5p/MAGEA3 axis	Cisplatin	[132]
TPTEP1	Downregulated	IL-6/STAT3 axis	Cisplatin	[133]
LINC00173	Upregulated	miR-641/RAB14 axis	Cisplatin	[139]
TINCR	Upregulated	miR-195-3p/ST6GAL1 axis	OXA	[135]
LINC01134	Upregulated	SP1/p62	OXA	[137]
CCAT1	Upregulated	QKI-5/p38 MAPK	OXA	[179]
HOTAIR	Upregulated	miR-34a/AKT axis	paclitaxel	[138]
LINC00680	Upregulated	miR-153-3p/AKT3 axis	5-FU	[139]
SNHG16	Upregulated	miR-93	5-FU	[141, 142]
AY	Upregulated	ITGAV	5-FU	[141, 142]
SNHG3	Upregulated	miR-214-3p/ASF1B axis	ICI	[144]
AC099850.3	Upregulated	PRR11/PI3K/AKT axis	ICI	[146]
LINC00244	Downregulated	PD-L1	ICI	[147]
HOXA-AS3	Upregulated	miR-455-5p/PD-L1 axis	ICI	[148]
LINC00638	Upregulated	miR-4732-3p/ULBP1 axis	ICI	[148]
CASC11	Upregulated	EIF4A3/E2F1/PD-L1 axis	ICI	[150]
MT1JP	Upregulated	BCL2L2	Lenvatinib	[180]
Hsa_Circ_0006988	Upregulated	IGF1	SOR	[181]
CircKCNN2	Downregulated	miR-520c-3p/MBD2	Lenvatinib	[158]
CircRNA-001241	Upregulated	miR-21-5p	SOR	[182]
CircMRPS35	Upregulated	miR-148a/ STX3	Cisplatin	[183]
CircMEMO1	Downregulated	TCF21	SOR	[184]

## Data Availability

No data was used for the research described in the article.

## References

[1] Forner A, Reig M, Bruix J (2018). Hepatocellular carcinoma. Lancet.

[2] Villanueva A (2019). Hepatocellular Carcinoma. N Engl J Med.

[3] Llovet JM (2021). Locoregional therapies in the era of molecular and immune treatments for hepatocellular carcinoma. Nat Rev Gastroenterol Hepatol.

[4] Yang JD, Heimbach JK (2020). New advances in the diagnosis and management of hepatocellular carcinoma. BMJ.

[5] Beaufrère A, Calderaro J, Paradis V (2021). Combined hepatocellular-cholangiocarcinoma: An update. J Hepatol.

[6] Zhong L (2021). Small molecules in targeted cancer therapy: advances, challenges, and future perspectives. Signal Transduct Target Ther.

[7] Tang W (2020). The mechanisms of sorafenib resistance in hepatocellular carcinoma: theoretical basis and therapeutic aspects. Signal Transduct Target Ther.

[8] Haider T (2020). Drug resistance in cancer: mechanisms and tackling strategies. Pharmacol Rep.

[9] Vasan N, Baselga J, Hyman DM (2019). A view on drug resistance in cancer. Nature.

[10] Li B (2020). Surmounting cancer drug resistance: New insights from the perspective of N(6)-methyladenosine RNA modification. Drug Resist Updat.

[11] Veldman J (2020). Primary and acquired resistance mechanisms to immune checkpoint inhibition in Hodgkin lymphoma. Cancer Treat Rev.

[12] Drago JZ, Modi S, Chandarlapaty S (2021). Unlocking the potential of antibody-drug conjugates for cancer therapy. Nat Rev Clin Oncol.

[13] Bukowski K, Kciuk M, Kontek R (2020). Mechanisms of multidrug resistance in cancer chemotherapy. Int J Mol Sci.

[14] Anastasiadou E, Jacob LS, Slack FJ (2018). Non-coding RNA networks in cancer. Nat Rev Cancer.

[15] Wang J (2019). ncRNA-encoded peptides or proteins and cancer. Mol Ther.

[16] To KK (2018). MicroRNAs in the prognosis and therapy of colorectal cancer: From bench to bedside. World J Gastroenterol.

[17] Xing C (2021). Role of lncRNA LUCAT1 in cancer. Biomed Pharmacother.

[18] Zhang HD (2018). CircRNA: a novel type of biomarker for cancer. Breast Cancer.

[19] Matsui M, Corey DR (2017). Non-coding RNAs as drug targets. Nat Rev Drug Discov.

[20] Slack FJ, Chinnaiyan AM (2019). The role of non-coding RNAs in oncology. Cell.

[21] Ghafouri-Fard S (2022). A review on the role of PCA3 lncRNA in carcinogenesis with an especial focus on prostate cancer. Pathol Res Pract.

[22] Fujita K, Nonomura N (2018). Urinary biomarkers of prostate cancer. Int J Urol.

[23] Guo S (2021). LncRNA PCA3 promotes antimony-induced lipid metabolic disorder in prostate cancer by targeting MIR-132-3 P/SREBP1 signaling. Toxicol Lett.

[24] Llovet JM (2018). Molecular therapies and precision medicine for hepatocellular carcinoma. Nat Rev Clin Oncol.

[25] Pan G (2021). EMT-associated microRNAs and their roles in cancer stemness and drug resistance. Cancer Commun (Lond).

[26] He B (2020). miRNA-based biomarkers, therapies, and resistance in Cancer. Int J Biol Sci.

[27] Li Y (2020). Bmi-1-induced miR-27a and miR-155 promote tumor metastasis and chemoresistance by targeting RKIP in gastric cancer. Mol Cancer.

[28] Li Y (2021). circRNA circARNT2 suppressed the sensitivity of hepatocellular carcinoma cells to cisplatin by targeting the miR-155-5p/PDK1 axis. Mol Ther Nucleic Acids.

[29] Van Roosbroeck K (2017). Combining anti-Mir-155 with chemotherapy for the treatment of lung cancers. Clin Cancer Res.

[30] Zhang L, Liao Y, Tang L (2019). MicroRNA-34 family: a potential tumor suppressor and therapeutic candidate in cancer. J Exp Clin Cancer Res.

[31] Jauhari A, Yadav S (2019). MiR-34 and MiR-200: regulator of cell fate plasticity and neural development. Neuromolecular Med.

[32] Welponer H (2020). The miR-34 family and its clinical significance in ovarian cancer. J Cancer.

[33] Naghizadeh S (2020). The role of miR-34 in cancer drug resistance. J Cell Physiol.

[34] Lu TX, Rothenberg ME (2018). MicroRNA. J Allergy Clin Immunol.

[35] Peng Y, Croce CM (2016). The role of MicroRNAs in human cancer. Signal Transduct Target Ther.

[36] Jia Y (2020). Roles of hsa-miR-12462 and SLC9A1 in acute myeloid leukemia. J Hematol Oncol.

[37] Pegtel DM, Gould SJ (2019). Exosomes. Annu Rev Biochem.

[38] Azmi AS, Bao B, Sarkar FH (2013). Exosomes in cancer development, metastasis, and drug resistance: a comprehensive review. Cancer Metastasis Rev.

[39] Wang W (2022). The potential roles of exosomal non-coding RNAs in hepatocellular carcinoma. Front Oncol.

[40] Álvarez-Garcia V (2019). Mechanisms of PTEN loss in cancer: It's all about diversity. Semin Cancer Biol.

[41] Fu X (2018). Exosomal microRNA-32-5p induces multidrug resistance in hepatocellular carcinoma via the PI3K/Akt pathway. J Exp Clin Cancer Res.

[42] Kong FH (2021). Current status of sorafenib nanoparticle delivery systems in the treatment of hepatocellular carcinoma. Theranostics.

[43] Yang J, Yan J, Liu B (2018). Targeting VEGF/VEGFR to modulate antitumor immunity. Front Immunol.

[44] Papadopoulos N, Lennartsson J (2018). The PDGF/PDGFR pathway as a drug target. Mol Aspects Med.

[45] Huang A (2020). Targeted therapy for hepatocellular carcinoma. Signal Transduct Target Ther.

[46] Cheng Z, Wei-Qi J, Jin D (2020). New insights on sorafenib resistance in liver cancer with correlation of individualized therapy. Biochim Biophys Acta Rev Cancer.

[47] Zhu AX (2010). Beyond sorafenib: novel targeted therapies for advanced hepatocellular carcinoma. Expert Opin Investig Drugs.

[48] Yaeger R, Corcoran RB (2019). Targeting alterations in the RAF-MEK pathway. Cancer Discov.

[49] El-Khoueiry AB (2021). Cabozantinib: an evolving therapy for hepatocellular carcinoma. Cancer Treat Rev.

[50] Ai L (2019). Sorafenib-associated hand-foot skin reaction: practical advice on diagnosis, mechanism, prevention, and management. Expert Rev Clin Pharmacol.

[51] Ladd AD (2023). Mechanisms of drug resistance in HCC. Hepatology.

[52] Ji L (2020). miR-486-3p mediates hepatocellular carcinoma sorafenib resistance by targeting FGFR4 and EGFR. Cell Death Dis.

[53] Pollutri D (2018). The epigenetically regulated miR-494 associates with stem-cell phenotype and induces sorafenib resistance in hepatocellular carcinoma. Cell Death Dis.

[54] Chen M (2022). miR-6071 inhibits hepatocellular carcinoma progression via targeting PTPN11. Arch Biochem Biophys.

[55] Wei Y (2022). miR-3154 promotes hepatocellular carcinoma progression via suppressing HNF4α. Carcinogenesis.

[56] He X (2021). Hsa-miR-4277 decelerates the metabolism or clearance of sorafenib in HCC cells and enhances the sensitivity of HCC cells to sorafenib by targeting cyp3a4. Front Oncol.

[57] Li TT (2021). MicroRNA-138-1-3p sensitizes sorafenib to hepatocellular carcinoma by targeting PAK5 mediated β-catenin/ABCB1 signaling pathway. J Biomed Sci.

[58] Feng X (2022). MiR-25 enhances autophagy and promotes sorafenib resistance of hepatocellular carcinoma via targeting FBXW7. Int J Med Sci.

[59] Enkhnaran B (2022). microRNA-106b-5p promotes cell growth and sensitizes chemosensitivity to sorafenib by targeting the BTG3/Bcl-xL/p27 signaling pathway in hepatocellular carcinoma. J Oncol.

[60] Zhang Z (2020). The miR-30a-5p/CLCF1 axis regulates sorafenib resistance and aerobic glycolysis in hepatocellular carcinoma. Cell Death Dis.

[61] Long HD (2018). Reduced hsa-miR-124-3p levels are associated with the poor survival of patients with hepatocellular carcinoma. Mol Biol Rep.

[62] Dong ZB (2022). MiRNA-124-3p.1 sensitizes hepatocellular carcinoma cells to sorafenib by regulating FOXO3a by targeting AKT2 and SIRT1. Cell Death Dis.

[63] Fornari F (2021). Elucidating the molecular basis of sorafenib resistance in HCC: current findings and future directions. J Hepatocell Carcinoma.

[64] Tan W (2021). miR-126-3p contributes to sorafenib resistance in hepatocellular carcinoma via downregulating SPRED1. Ann Transl Med.

[65] Hu X (2021). The role of non-coding RNAs in the sorafenib resistance of hepatocellular carcinoma. Front Oncol.

[66] Wang N (2018). microRNA-23a in human cancer: its roles, mechanisms and therapeutic relevance. Cancers (Basel).

[67] Lu Y (2022). Epigenetic regulation of ferroptosis via ETS1/miR-23a-3p/ACSL4 axis mediates sorafenib resistance in human hepatocellular carcinoma. J Exp Clin Cancer Res.

[68] Xi J, Ma CX (2020). Sequencing endocrine therapy for metastatic breast cancer: what do we do after disease progression on a CDK4/6 inhibitor?. Curr Oncol Rep.

[69] Saini N (2020). Mutation signatures specific to DNA alkylating agents in yeast and cancers. Nucleic Acids Res.

[70] Stupp R (2017). Effect of tumor-treating fields plus maintenance temozolomide vs maintenance temozolomide alone on survival in patients with glioblastoma: a randomized clinical trial. JAMA.

[71] Gurjao C (2021). Discovery and features of an alkylating signature in colorectal cancer. Cancer Discov.

[72] Wei L (2020). Noncoding RNAs in gastric cancer: implications for drug resistance. Mol Cancer.

[73] Wang H (2022). Long non-coding RNA placenta-specific protein 2 regulates the chemosensitivity of cancer cells to cisplatin in hepatocellular carcinoma (HCC) by sponging microRNA-96 to upregulate X-linked inhibitor of apoptosis protein. Bioengineered.

[74] Meng X (2021). The role of non-coding RNAs in drug resistance of oral squamous cell carcinoma and therapeutic potential. Cancer Commun (Lond).

[75] Yang Y (2021). Biosci Rep.

[76] Fang D (2021). Binding features and functions of ATG3. Front Cell Dev Biol.

[77] Zou L, Sun P, Zhang L (2021). miR-651-3p enhances the sensitivity of hepatocellular carcinoma to cisplatin via targeting ATG3-mediated cell autophagy. J Oncol.

[78] Zeng T (2021). Upregulation of miR-138 increases sensitivity to cisplatin in hepatocellular carcinoma by regulating EZH2. Biomed Res Int.

[79] Bao Y (2020). Overexpression of microRNA-9 enhances cisplatin sensitivity in hepatocellular carcinoma by regulating EIF5A2-mediated epithelial-mesenchymal transition. Int J Biol Sci.

[80] Xu Y, Wang H, Gao W (2020). MiRNA-610 acts as a tumour suppressor to depress the cisplatin resistance in hepatocellular carcinoma through targeted silencing of hepatoma-derived growth factor. Arch Med Sci.

[81] Chen S (2019). miR-21-5p suppressed the sensitivity of hepatocellular carcinoma cells to cisplatin by targeting FASLG. DNA Cell Biol.

[82] Lin CC (2020). Safety and preliminary efficacy of ramucirumab in combination with FOLFOX4 in patients with advanced hepatocellular carcinoma: a nonrandomized, open-label, Phase IB Study. Oncologist.

[83] Li M (2021). Cost-effectiveness analysis of hepatic arterial infusion of FOLFOX combined sorafenib for advanced hepatocellular carcinoma with portal vein invasion. Front Oncol.

[84] Ren WW (2018). MicroRNA-125b reverses oxaliplatin resistance in hepatocellular carcinoma by negatively regulating EVA1A mediated autophagy. Cell Death Dis.

[85] Cao F, Yin LX (2019). miR-122 enhances sensitivity of hepatocellular carcinoma to oxaliplatin via inhibiting MDR1 by targeting Wnt/β-catenin pathway. Exp Mol Pathol.

[86] Schwabe RF, Greten TF (2020). Gut microbiome in HCC—mechanisms, diagnosis and therapy. J Hepatol.

[87] Eyler RF, Shvets K (2019). Clinical pharmacology of antibiotics. Clin J Am Soc Nephrol.

[88] Xie C (2020). A hMTR4-PDIA3P1-miR-125/124-TRAF6 regulatory axis and its function in nf kappa B signaling and chemoresistance. Hepatology.

[89] Duan H (2021). Recent advances in drug delivery systems for targeting cancer stem cells. Acta Pharm Sin B.

[90] Wang J (2020). MicroRNA-135a promotes proliferation, migration, invasion and induces chemoresistance of endometrial cancer cells. Eur J Obstet Gynecol Reprod Biol X.

[91] Wei XC (2021). Hepatitis B core antigen modulates exosomal miR-135a to target vesicle-associated membrane protein 2 promoting chemoresistance in hepatocellular carcinoma. World J Gastroenterol.

[92] Chen E (2021). miR-26b enhances the sensitivity of hepatocellular carcinoma to Doxorubicin via USP9X-dependent degradation of p53 and regulation of autophagy. Int J Biol Sci.

[93] Cui X (2020). MicroRNA200a enhances antitumor effects in combination with doxorubicin in hepatocellular carcinoma. Transl Oncol.

[94] Magadum A (2020). Pkm2 regulates cardiomyocyte cell cycle and promotes cardiac regeneration. Circulation.

[95] Liu L (2019). The cell cycle in stem cell proliferation, pluripotency and differentiation. Nat Cell Biol.

[96] Zhu L, Chen L (2019). Progress in research on paclitaxel and tumor immunotherapy. Cell Mol Biol Lett.

[97] Barata PC, Sartor AO (2019). Metastatic castration-sensitive prostate cancer: abiraterone, docetaxel, or…. Cancer.

[98] Shanbhag S, Ambinder RF (2018). Hodgkin lymphoma: a review and update on recent progress. CA Cancer J Clin.

[99] Wang Y (2021). The role of non-coding RNAs in ABC transporters regulation and their clinical implications of multidrug resistance in cancer. Expert Opin Drug Metab Toxicol.

[100] Abu Samaan TM (2019). Paclitaxel's mechanistic and clinical effects on breast cancer. Biomolecules.

[101] Huang Y (2018). Inhibition of microRNA-16 facilitates the paclitaxel resistance by targeting IKBKB via NF-κB signaling pathway in hepatocellular carcinoma. Biochem Biophys Res Commun.

[102] Yang J, Cui R, Liu Y (2020). MicroRNA-212-3p inhibits paclitaxel resistance through regulating epithelial-mesenchymal transition, migration and invasion by targeting ZEB2 in human hepatocellular carcinoma. Oncol Lett.

[103] Lyu N (2022). Arterial chemotherapy of oxaliplatin plus fluorouracil versus sorafenib in advanced hepatocellular carcinoma: a biomolecular exploratory, randomized, phase III trial (FOHAIC-1). J Clin Oncol.

[104] Sethy C, Kundu CN (2021). 5-Fluorouracil (5-FU) resistance and the new strategy to enhance the sensitivity against cancer: implication of DNA repair inhibition. Biomed Pharmacother.

[105] Siddiqui NS (2019). Capecitabine for the treatment of pancreatic cancer. Expert Opin Pharmacother.

[106] Pinyopornpanish K (2021). Chemopreventive effect of statin on hepatocellular carcinoma in patients with nonalcoholic steatohepatitis cirrhosis. Am J Gastroenterol.

[107] Sidaway P (2022). FOLFOX-HAIC active in large HCC. Nat Rev Clin Oncol.

[108] Goyal L (2019). A phase ii and biomarker study of sorafenib combined with modified FOLFOX in patients with advanced hepatocellular carcinoma. Clin Cancer Res.

[109] Vodenkova S (2020). 5-fluorouracil and other fluoropyrimidines in colorectal cancer: past, present and future. Pharmacol Ther.

[110] Shao P (2017). MicroRNA-205-5p regulates the chemotherapeutic resistance of hepatocellular carcinoma cells by targeting PTEN/JNK/ANXA3 pathway. Am J Transl Res.

[111] Zheng RP (2020). MiR-145 regulates the chemoresistance of hepatic carcinoma cells against 5-fluorouracil by targeting toll-like receptor 4. Cancer Manag Res.

[112] Jiang Y (2019). PD-1 and PD-L1 in cancer immunotherapy: clinical implications and future considerations. Hum Vaccin Immunother.

[113] Ai L, Xu A, Xu J (2020). Roles of PD-1/PD-L1 pathway: signaling, cancer, and beyond. Adv Exp Med Biol.

[114] Rowshanravan B, Halliday N, Sansom DM (2018). CTLA-4: a moving target in immunotherapy. Blood.

[115] Bu MT (2022). The roles of TGF-β and VEGF pathways in the suppression of antitumor immunity in melanoma and other solid tumors. Pharmacol Ther.

[116] Tie Y (2022). Immunosuppressive cells in cancer: mechanisms and potential therapeutic targets. J Hematol Oncol.

[117] Adams S (2019). Pembrolizumab monotherapy for previously untreated, PD-L1-positive, metastatic triple-negative breast cancer: cohort B of the phase II KEYNOTE-086 study. Ann Oncol.

[118] Hamid O (2019). Five-year survival outcomes for patients with advanced melanoma treated with pembrolizumab in KEYNOTE-001. Ann Oncol.

[119] Nowicki TS, Hu-Lieskovan S, Ribas A (2018). Mechanisms of resistance to PD-1 and PD-L1 blockade. Cancer J.

[120] Iqbal MA (2019). MicroRNA in lung cancer: role, mechanisms, pathways and therapeutic relevance. Mol Aspects Med.

[121] Zhang G (2015). microRNA-4717 differentially interacts with its polymorphic target in the PD1 3' untranslated region: a mechanism for regulating PD-1 expression and function in HBV-associated liver diseases. Oncotarget.

[122] Liu J (2019). Endoplasmic reticulum stress causes liver cancer cells to release exosomal miR-23a-3p and Up-regulate programmed death ligand 1 expression in macrophages. Hepatology.

[123] Bridges MC, Daulagala AC, Kourtidis A (2021). LNCcation: lncRNA localization and function. J Cell Biol.

[124] Peng WX, Koirala P, Mo YY (2017). LncRNA-mediated regulation of cell signaling in cancer. Oncogene.

[125] Tan YT (2021). LncRNA-mediated posttranslational modifications and reprogramming of energy metabolism in cancer. Cancer Commun (Lond).

[126] Cabral LKD, Tiribelli C, Sukowati CHC (2020). Sorafenib resistance in hepatocellular carcinoma: the relevance of genetic heterogeneity. Cancers (Basel).

[127] Zheng ZQ (2019). Long noncoding RNA FAM225A promotes nasopharyngeal carcinoma tumorigenesis and metastasis by acting as ceRNA to sponge miR-590-3p/miR-1275 and upregulate ITGB3. Cancer Res.

[128] Liu YT, Liu GQ, Huang JM (2020). Biosci Rep.

[129] Zhang J (2020). KCNQ1OT1 contributes to sorafenib resistance and programmed death-ligand-1-mediated immune escape via sponging miR-506 in hepatocellular carcinoma cells. Int J Mol Med.

[130] Chen YT (2021). Upregulation of lncRNA NIFK-AS1 in hepatocellular carcinoma by m(6)A methylation promotes disease progression and sorafenib resistance. Hum Cell.

[131] Shi Y (2020). HANR enhances autophagy-associated sorafenib resistance through miR-29b/ATG9A axis in hepatocellular carcinoma. Onco Targets Ther.

[132] Chen Y (2020). LINC01234/MicroRNA-31-5p/MAGEA3 axis mediates the proliferation and chemoresistance of hepatocellular carcinoma cells. Mol Ther Nucleic Acids.

[133] Ding H (2019). Long non-coding RNA TPTEP1 inhibits hepatocellular carcinoma progression by suppressing STAT3 phosphorylation. J Exp Clin Cancer Res.

[134] Zhao G (2021). Long non-coding RNA LINC00173 enhances cisplatin resistance in hepatocellular carcinoma via the microRNA-641/RAB14 axis. Oncol Lett.

[135] Mei J (2022). Long noncoding RNA TINCR facilitates hepatocellular carcinoma progression and dampens chemosensitivity to oxaliplatin by regulating the miR-195-3p/ST6GAL1/NF-κB pathway. J Exp Clin Cancer Res.

[136] Kang X (2022). Silenced LINC01134 enhances oxaliplatin sensitivity by facilitating ferroptosis through GPX4 in hepatocarcinoma. Front Oncol.

[137] Ma L (2021). LSD1-demethylated LINC01134 confers oxaliplatin resistance through SP1-induced p62 transcription in HCC. Hepatology.

[138] Duan Y (2020). Biosci Rep.

[139] Yang Y (2021). Long non-coding RNA FGD5-AS1 contributes to cisplatin resistance in hepatocellular carcinoma via sponging microRNA-153-3p by upregulating Twinfilin Actin Binding Protein 1 (TWF1). Bioengineered.

[140] Shu G (2021). LINC00680 enhances hepatocellular carcinoma stemness behavior and chemoresistance by sponging miR-568 to upregulate AKT3. J Exp Clin Cancer Res.

[141] Xu F (2018). Overexpressing lncRNA SNHG16 inhibited HCC proliferation and chemoresistance by functionally sponging hsa-miR-93. Onco Targets Ther.

[142] Kang CL (2019). LncRNA AY promotes hepatocellular carcinoma metastasis by stimulating ITGAV transcription. Theranostics.

[143] Shi F (2011). PD-1 and PD-L1 upregulation promotes CD8(+) T-cell apoptosis and postoperative recurrence in hepatocellular carcinoma patients. Int J Cancer.

[144] Segura-Bayona S, Stracker TH (2019). The Tousled-like kinases regulate genome and epigenome stability: implications in development and disease. Cell Mol Life Sci.

[145] Zhan T (2021). Construction of novel lncRNA-miRNA-mRNA network associated with recurrence and identification of immune-related potential regulatory axis in hepatocellular carcinoma. Front Oncol.

[146] Zhong F (2022). LncRNA AC099850.3 promotes hepatocellular carcinoma proliferation and invasion through PRR11/PI3K/AKT axis and is associated with patients prognosis. J Cancer.

[147] Sun Z (2022). LINC00244 suppresses cell growth and metastasis in hepatocellular carcinoma by downregulating programmed cell death ligand 1. Bioengineered.

[148] Zeng C (2021). HOXA-AS3 promotes proliferation and migration of hepatocellular carcinoma cells via the miR-455-5p/PD-L1 axis. J Immunol Res.

[149] Qi F (2021). Tumor mutation burden-associated LINC00638/miR-4732-3p/ULBP1 axis promotes immune escape via PD-L1 in hepatocellular carcinoma. Front Oncol.

[150] Song H (2020). Long noncoding RNA CASC11 promotes hepatocarcinogenesis and HCC progression through EIF4A3-mediated E2F1 activation. Clin Transl Med.

[151] Zhou WY (2020). Circular RNA: metabolism, functions and interactions with proteins. Mol Cancer.

[152] Du WW (2017). Identifying and characterizing circRNA-protein interaction. Theranostics.

[153] Jiang W (2021). The role of lncRNAs and circRNAs in the PD-1/PD-L1 pathway in cancer immunotherapy. Mol Cancer.

[154] Liu L (2020). Noncoding RNAs: the shot callers in tumor immune escape. Signal Transduct Target Ther.

[155] Tokunaga R (2018). CXCL9, CXCL10, CXCL11/CXCR3 axis for immune activation—a target for novel cancer therapy. Cancer Treat Rev.

[156] Huang XY (2020). Circular RNA circMET drives immunosuppression and anti-PD1 therapy resistance in hepatocellular carcinoma via the miR-30-5p/snail/DPP4 axis. Mol Cancer.

[157] Zhang PF (2020). Cancer cell-derived exosomal circUHRF1 induces natural killer cell exhaustion and may cause resistance to anti-PD1 therapy in hepatocellular carcinoma. Mol Cancer.

[158] Liu D (2022). circKCNN2 suppresses the recurrence of hepatocellular carcinoma at least partially via regulating miR-520c-3p/methyl-DNA-binding domain protein 2 axis. Clin Transl Med.

[159] Gogry FA (2021). Current update on intrinsic and acquired colistin resistance mechanisms in bacteria. Front Med (Lausanne).

[160] Li Y (2022). Nucleic acid therapy in pediatric cancer. Pharmacol Res.

[161] Saw PE, Song EW (2020). siRNA therapeutics: a clinical reality. Sci China Life Sci.

[162] Crooke ST (2018). RNA-targeted therapeutics. Cell Metab.

[163] Samaridou E, Heyes J, Lutwyche P (2020). Lipid nanoparticles for nucleic acid delivery: Current perspectives. Adv Drug Deliv Rev.

[164] Gupta A (2021). Nucleic acid delivery for therapeutic applications. Adv Drug Deliv Rev.

[165] Kulkarni JA (2021). The current landscape of nucleic acid therapeutics. Nat Nanotechnol.

[166] Pereira-Silva M (2020). Micelleplexes as nucleic acid delivery systems for cancer-targeted therapies. J Control Release.

[167] Barbieri I, Kouzarides T (2020). Role of RNA modifications in cancer. Nat Rev Cancer.

[168] Giri P (2021). Chemical modification of enzymes to improve biocatalytic performance. Biotechnol Adv.

[169] Shao YY (2022). Low miR-10b-3p associated with sorafenib resistance in hepatocellular carcinoma. Br J Cancer.

[170] Zhang Y (2021). Inhibition of miR-15a-5p promotes the chemoresistance to pirarubicin in hepatocellular carcinoma via targeting eIF4E. Comput Math Methods Med.

[171] Yang D (2022). miR-4461 inhibits liver cancer stem cells expansion and chemoresistance via regulating SIRT1. Carcinogenesis.

[172] Cheng Z (2021). MicroRNA-92b augments sorafenib resistance in hepatocellular carcinoma via targeting PTEN to activate PI3K/AKT/mTOR signaling. Braz J Med Biol Res.

[173] Li X (2023). Silencing lncRNA HCG18 regulates GPX4-inhibited ferroptosis by adsorbing miR-450b-5p to avert sorafenib resistance in hepatocellular carcinoma. Hum Exp Toxicol.

[174] Kong H (2022). Long intergenic non-protein coding RNA 1273 confers sorafenib resistance in hepatocellular carcinoma via regulation of methyltransferase 3. Bioengineered.

[175] Lin JC, Yang PM, Liu TP (2021). PERK/ATF4-dependent ZFAS1 upregulation is associated with sorafenib resistance in hepatocellular carcinoma cells. Int J Mol Sci.

[176] Sun J (2022). LncRNA LIMT (LINC01089) contributes to sorafenib chemoresistance via regulation of miR-665 and epithelial to mesenchymal transition in hepatocellular carcinoma cells. Acta Biochim Biophys Sin (Shanghai).

[177] Berhane T (2022). Knockdown of the long noncoding RNA PURPL induces apoptosis and sensitizes liver cancer cells to doxorubicin. Sci Rep.

[178] Li Y (2022). The lncARSR/PTEN/Akt/nuclear factor-kappa B feedback regulatory loop contributes to doxorubicin resistance in hepatocellular carcinoma. J Biochem Mol Toxicol.

[179] Xia C (2022). LncRNA CCAT1 enhances chemoresistance in hepatocellular carcinoma by targeting QKI-5. Sci Rep.

[180] Yu T (2021). MT1JP-mediated miR-24-3p/BCL2L2 axis promotes Lenvatinib resistance in hepatocellular carcinoma cells by inhibiting apoptosis. Cell Oncol (Dordr).

[181] Qiu R, Zeng Z (2022). Hsa_circ_0006988 promotes sorafenib resistance of hepatocellular carcinoma by modulating IGF1 using miR-15a-5p. Can J Gastroenterol Hepatol.

[182] Yang Q, Wu G (2022). CircRNA-001241 mediates sorafenib resistance of hepatocellular carcinoma cells by sponging miR-21-5p and regulating TIMP3 expression. Gastroenterol Hepatol.

[183] Li P (2022). circMRPS35 promotes malignant progression and cisplatin resistance in hepatocellular carcinoma. Mol Ther.

[184] Dong ZR (2021). CircMEMO1 modulates the promoter methylation and expression of TCF21 to regulate hepatocellular carcinoma progression and sorafenib treatment sensitivity. Mol Cancer.

